# Latent encoding of movement in primary visual cortex

**DOI:** 10.1371/journal.pcbi.1014826

**Published:** 2026-10-12

**Authors:** Hannah Germaine, Isaiah Choi, Stephen D. Van Hooser, Jérôme Ribot, Jonathan David Touboul

**Affiliations:** 1 Department of Mathematics, Brandeis University, Waltham, Massachusetts, United States of America; 2 Department of Biology, Brandeis University, Waltham, Massachusetts, United States of America; 3 Volen National Center for Complex Systems, Brandeis University, Waltham, Massachusetts, United States of America; 4 Neuroglial Interactions in Cerebral Physiology and Pathologies, Center for Interdisciplinary Research in Biology, Collège de France, CNRS, INSERM, Labex Memolife, Université PSL, Paris, France; Université Paris Cité: Universite Paris Cite, FRANCE

## Abstract

Neurons in the primary visual cortex (V1) are classically thought to encode spatial features of visual stimuli through simple population codes: each neuron exhibits a preferred orientation and preferred spatial frequency, both of which remaining invariant to other aspects of the visual stimulus. Here, we show that this simple rule does not apply to the representation of major features of stimulus motion, including stimulus direction and temporal frequency (TF). We collected an extensive dataset of cat V1 responses to stimuli covarying in orientation, direction, spatial frequency, and TF to assess the extent of motion selectivity. We show that preferred TF is mostly uniform across the cortical surface. Yet, in over half of V1, the preferred direction is reversed with changing stimulus TF, revealing four distinct map motifs embedded in V1’s functional architecture. Similarly, despite the lack of spatial modulation for the preferred TF map and the lack of invariance for the preferred direction map, we found using convolutional neural networks that direction, TF and stimulus speed can be accurately decoded from V1 responses at all cortical locations. These findings suggest a latent encoding of information where subtle, spatially distributed modulations of V1 activity may convey fine information about stimulus motion, pointing to a novel primary sensory encoding mechanism despite complex co-variation of responses to multiple attributes across V1 neurons.

## Author summary

Understanding how the external world is represented in the brain has long been a central endeavor in neuroscience. In the mammalian primary visual cortex (V1), groups of neurons encode stimulus properties through changes in activity, with “preferred” stimuli eliciting maximal responses, as demonstrated for orientation and spatial frequency. We collected an extensive dataset of neuronal responses to investigate whether V1 also encodes motion speed. We found that cortical response organization remains largely invariant, in the sense that neurons uniformly exhibit a preference to the same temporal frequency. Yet, machine learning algorithms decode motion speed with remarkable precision, revealing that subtle, spatially distributed modulations of activity underlie speed encoding. Moreover, we show that preferred direction flips with speed across 80% of cortex, uncovering a novel co-organization of motion and direction information in V1.

## Introduction

Motion perception, the ability to infer the speed and direction of motion of elements in a visual scene is a central capacity of the mammalian visual system and essential for animal survival. In dynamic visual scenes, motion is characterized by local directions and velocities of points or edges in space, experimentally studied using spatio-temporal gratings characterized by a *speed* (in degrees of visual angle per second) given by the ratio of stimulus Temporal Frequency (TF, in Hz) to Spatial Frequency (SF, in cycles/degree).

The neural mechanisms underlying motion perception have been a central topic of study in neuroscience [1]. The primary visual cortex (V1) has been deemed to have a central role in motion processing since the pioneering works of Hubel and Wiesel [2] that identified direction-selective neurons in cat V1, later confirmed in primates [3,4], cats [5,6], ferrets [7–10], and mice [11].

Local components of motion processed in V1 are then transmitted to higher visual areas, such as the middle temporal area (MT, or V5) in Human or Macaque monkey, for further integration [9,12–14]. MT allows for low-speed detection of objects, and evidence in Human suggests that another pathway bypassing V1 brings information directly from the lateral geniculate nucleus for processing fast-moving stimuli [15,16]. Notably, neurons in MT in macaque are highly specialized for motion processing, responding robustly to moving stimuli to produce a coherent representation of complex motion patterns across the visual field. Moreover, these neurons are highly selective for speed [17–20], but do not cluster into functional columns with similar speed tuning [17].

This speed selectivity of MT neurons contrasts with V1, where experimental data present a more complex picture. Studies reported speed- or TF-tuned neurons in macaque V1 [3,4,21]. V1 neurons also reportedly exhibit a continuum of spatiotemporal tuning properties ranging from separable TF/SF tuning, where preferred TF remains largely independent of SF, to partial or near speed tuning, where preferred speed remains more stable according to the ratio TF/SF [3,4,21]. Such responses are classically interpreted within spatiotemporal models of motion processing, in which motion selectivity emerges from filtering in space-time and pooling across spatiotemporal frequency domains [22,23]. In human V1, studies reported that SF and TF preferences cluster spatially in a binary co-representation [24]. Similar results have been found in mouse V1 [25] or cat [26], where cells also exhibit a continuum from speed to TF selectivity [11], while uniform representation of TF was found in the bush baby (*Otolemur garnetti*) [27].

To further add complexity to the understanding of SF, TF, and speed tuning in V1, evidence from electrophysiological studies in cats [28] and ferrets [7] have suggested that while preferred orientation remains invariant to changes in TF, direction selectivity can vary significantly. In ferret V1, it has been shown that direction preference changes with TF, while orientation tuning remains stable across a range of temporal frequencies [7]. Similarly, in the cat visual cortex, direction selectivity is strongest at low temporal frequencies (1–2 Hz) but diminishes or even reverses at higher temporal frequencies [28]. These findings suggest that TF/speed maps and direction maps could co-vary and should thus be studied together to fully understand how motion is encoded in V1.

In this study, we investigate whether similar phenomena arise at a mesoscopic level. We study speed selectivity in cat V1 (area 17) using high-resolution intrinsic optical imaging. Leveraging recent advancements in stimulation techniques and analysis methods [29–31], we collected an extensive dataset of V1 responses to a variety of stimuli changing in orientation, direction, SF and TF. We examine how functional maps of orientation and direction varied across 45 combinations of SF and TF (5 SF× 9 TF). We next explore the information contained in V1’s activity patterns. In more detail, we investigate whether population activity patterns carry motion information in the absence of a structured map? To probe this, we use statistical machine learning algorithms and convolutional neural networks to decode the stimulus properties, a methodology comparable to the more classical Multi-Voxel Pattern Analysis in fMRI studies [32–34]. This allows us to demonstrate that spatially distributed V1 activity patterns contain fine local information about the direction and speed of motion. This methodology, akin to Additionally, while changing stimulus TF leaves orientation maps unaltered, we found a significant remapping of the preferred direction maps, with smooth variations of the position of the direction fractures with occasional singular motifs forming bubbles or saddles. Similar to single cell studies, these maps show a maximal invariance along an axis corresponding to a power-law relationship between SF and TF, with an exponent intermediate between separable SF-TF tuning and speed tuning. Altogether, these findings reveal a latent encoding of the movement information in the spatially distributed activity in V1 that does not affect activity profiles and orientation responses, but does impact direction representation, providing novel insights into the neural encoding of complex motion dynamics in cat primary visual cortex.

## Materials and methods

### Ethics statement

All experiments were performed in accordance with the relevant institutional and national guidelines and regulations from Collège de France, CNRS and French Direction départementale de la protection des populations (DDPP, JO 87–848, consolidated after revision on May 30, 2001, Certificate no. 75–1754, French “Ministère de l’Agriculture et de la Pêche”). They are also in compliance with the relevant regulatory standards recommended by the European Community Directive (Directive 2010/63/UE) and the US National Institutes of Health.

### Surgical procedure

Three young adult cats (1 male, 2 females), aged 8–11 weeks and weighing 1.1–1.6 kg, were used in this study. All animals were in good health and with no apparent malformations or pathologies. Animals were anesthetized with saffan (initial dose, 1.2 mg/kg, i.m.; supplements, 1:1 in saline, administered intravenously as needed). Following tracheal and venous cannulation, electrocardiogram, temperature, and expired CO2 probes were placed for continuous monitoring and animals were secured in the Horsley-Clarke stereotactic frame in preparation for acute recordings. The scalp was incised in the sagittal plane, and a large craniotomy was performed over area 17. The nictitating membranes were then retracted with eye drops (Neo-Synephrine 5%, Ciba Vision Ophthalmics), and the pupils were dilated with atropine eye drops (atropine 1%, MSD-Chibret). Scleral lenses were placed to protect the cornea and focus the eyes on the tangent screen 28.5 cm away. Animals were then paralyzed with an infusion of Pavulon (0.2 mL/kg (0.4 mg/kg), i.v.), and breathing was assisted artificially through a tracheal cannula with a 3:2 mixture of N2O and O2 containing 0.5-1.0% isoflurane. The respiration frequency was adjusted to 18 breaths/minute, and the volume adapted to the ratio of exhaled CO2 (partial pressure of carbon dioxide at 4%). Paralysis was maintained throughout the recording by continuous infusion of a mixture of Pavulon (0.1 mL/kg/h) diluted in glucose (5%) and NaCl (0.9g/L). Following the recording session, animals were sacrificed using a lethal dose of pentobarbital.

### Optical imaging

The cortex was illuminated at 545 nm to reveal the vascular pattern of the cortical surface, and at 700 nm to record the intrinsic signals. The focal plane was adjusted to 500 μm below the cortical surface. The optic discs were plotted by tapetal reflection on a sheet of paper covering the tangent screen placed 28.5 cm in front of the animal. The center of the screen was situated 8 cm below the middle of the two optic discs. Intrinsic optical signals were recorded while the animals were exposed to visual stimuli displayed on a CRT monitor subtending a visual angle of around $75^{\circ }\times 56^{\circ }$. Frames were acquired by CCD video camera (1M60, DALSA) at the rate of 40 frames per second and were stored after binning by 2x2 pixels spatially and by 12 frames temporally using the LongDaq system (Optical Imaging). Images were acquired with a resolution of 12.3 μm/pixel.

### Stimulation

Full-screen visual stimuli were presented continuously to the animal. In order to sample a large number of stimulus conditions within a single recording session, we used continuous rotating drifting gratings, a stimulation paradigm that allows the acquisition of a complete direction map in approximately 10 minutes [30,31]. Each stimulus consisted of sine-wave gratings drifting in one direction and rotating in a counter-clockwise manner. The angular speed of rotation was one cycle per minute. The contrast was set at 50% to ensure the production of smooth sine wave gratings [35]. Nine temporal frequencies ranging in a logarithmic scale from 0.38 to 5.37 Hz, and 5 spatial frequencies ranging in a logarithmic scale from 0.15 to 1.65 cycles/degree were presented in random order. Ten full rotations were presented for each pair of temporal and spatial frequencies. Stimuli were presented binocularly to avoid extending the total acquisition time (~ 7 hours) and to maintain the physiological stability of the preparation. The center of rotation of the stimulus was the center of the visual display. The impact of the stimulus rotation was minimal relative to the range of grating drift speeds tested: its impact, increasing with eccentricity, reached only speeds of $\sim 4.9^{\circ }/s$ at the corners of the display and thus may produce a slight shift in speed correspondence away from the central region.

### Image processing

To ensure that the data is recorded from area 17, we relied on a functional criterion based on spatial frequency sensitivity: data from A18 were rejected from further analysis based on responses evoked by low spatial frequency stimuli (0.15 cpd, optimal in A18) versus higher spatial frequency stimuli (0.5 cpd, optimal in A17).

Data analysis was based on the method developed by Kalatsky and Stryker [30,31] to extract periodic signals from intrinsic data using Fourier decompositions. For each SF and TF, data were preprocessed to remove slow-varying components using a multivariate analysis technique, the generalized indicator function method [36,37]. To correct for the delays and amplitude attenuation related to the filtering associated with hemodynamic signals and accurately recover stimulus-locked activity, we applied a frequency-domain inverse filter based on a first-order low-pass model with a 5-second time constant [30]. After this deconvolution, the resulting activity was smoothed using a low-pass spatial filter (Gaussian kernel of 5x5 pixels and a 2-pixel standard deviation). These operations resulted, for each SF and TF, in maps of the changes in reflectance according to direction, noted ΔI. From these signals, we computed the *rescaled signals*, that are linear transformations of the signal mapping responses to the interval [0,1], and obtained from the reflectance signal at a given condition, by subtracting the lowest value, and then diving this by the maximal of this new response map. Rescaled signals conserve the relative changes in amplitude but assure that maps have the same amplitudes and range between 0 and 1.To smooth individual pixel responses in Fig 1G, we used a median kernel on SF, TF and direction, where the median was taken on neighboring TF and SF, and neighboring direction in a cone of 25 degrees of opening.

**Fig 1:**
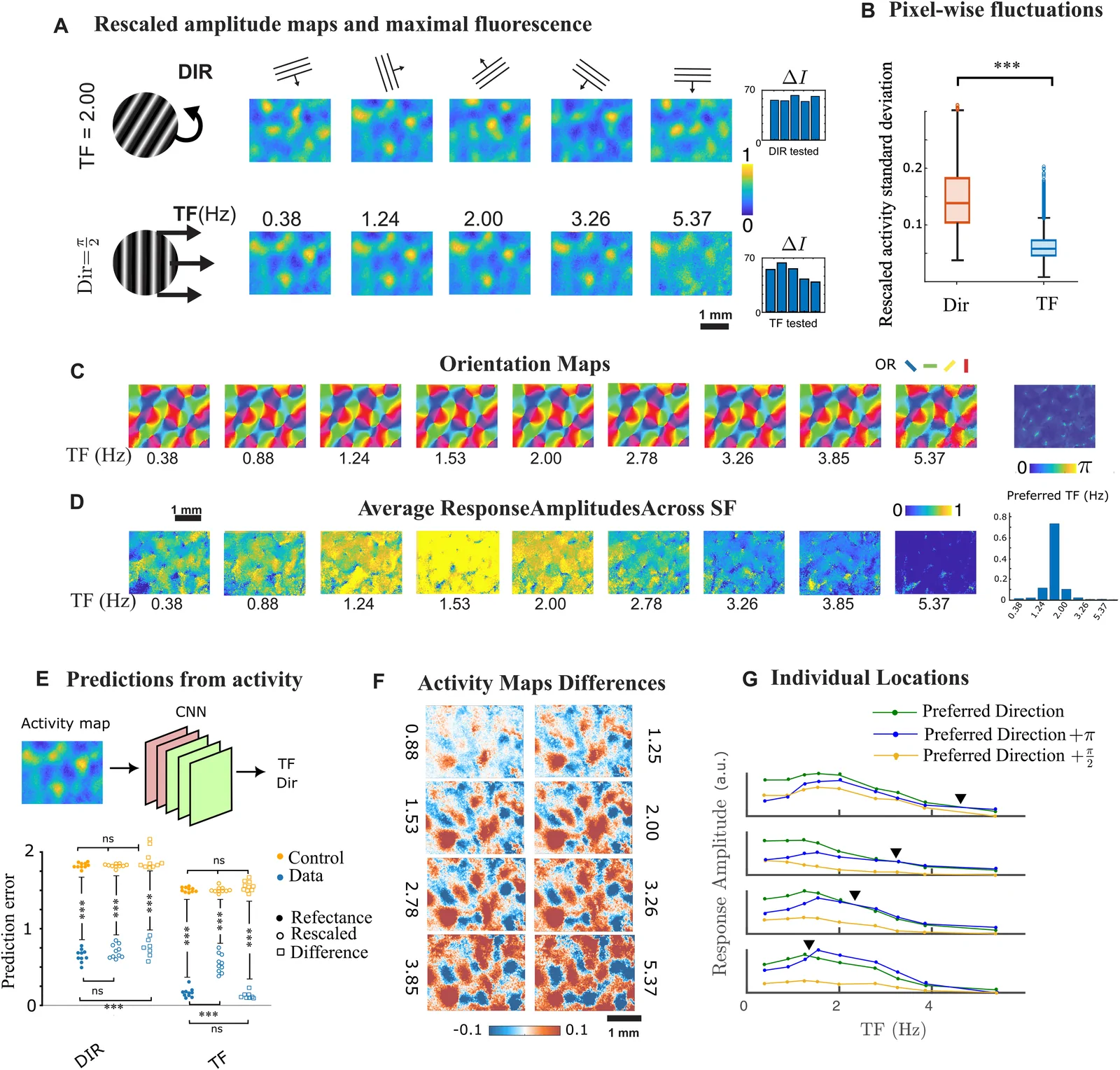
Uniform representation of temporal frequency preference with decodable information. A-D: Invariant features. **A.** Top: Response amplitude ΔI (measured as a change in reflectance, see Methods) for various directions of motion and a fixed TF (2 Hz). Bottom: Response amplitude for various TFs and a fixed direction (rightward, π/2) (bottom). In both panels, the stimulus spatial frequency is 0.29 cycles/degree. The responses are rescaled between 0 and 1. The mean response amplitude for each stimulus condition is plotted on the right. **B.** Standard deviation of the response magnitude at each pixel when varying stimulus direction and stimulus TF. Median standard deviation for direction is 0.11±0.04 mad, while for TF it is 0.03±0.03 mad (two-sample t-test *p* < 0.001). **C.** Orientation maps are largely invariant to changes in stimulus TF (median standard deviation of 0.2±0.15 mad radian). Right: standard deviation of the response for each pixel, with higher fluctuations near orientation pinwheel centers. Right: Pixel-wise standard deviation of the orientation preference across TF conditions. Higher fluctuations are observed near orientation pinwheel centers. **D.** Amplitude of responses to changes in TF, averaged across SF at preferred orientation. The preferred TF shows a unimodal distribution (histogram on the right) that peaks at 1.53 Hz, with 90% of locations within the 1.24-2.00 Hz range (cat 2: 81% and cat 3: 78%). **E.** Top: The architecture of the Convolutional Neural Network (CNN) is composed of five layers (two convolutional in pink and three rectified linear layers in green). This CNN was trained to predict stimulus TF and direction from V1 responses. Bottom: Prediction error (blue dots) shows high predictability for TF and direction compared to controls (same dataset but where TF and Direction were randomly shuffled, yellow dots) (10-fold cross-validation, two-sample t-test *p* < 0.001). **F.** Difference between V1 response maps between *TF* = 0.38 and other stimulus TFs. These differences show a gradual increase in strength with increasing TFs, as well as smooth variations in spatial organization. Stimulus spatial frequency is *SF* = 0.29 cycles/degree and stimulus direction π/2. **G.** Smoothed responses of four cortical locations separated by 87μm along a line, showing changes in response with varying stimulus TF. The preferred direction was determined at TF = 0.38 Hz, and response tuning curves are displayed for the preferred direction (green), the opposite direction (blue) and the orthogonal direction (orange). Black triangles indicate switches in preferred direction for each cortical location.

To calculate orientation and direction maps, we first extracted the Fourier coefficients at the frequency of stimulation (fundamental frequency, or first harmonic H1) and the second harmonic (H2) of the angular component of the response at each pixel, for each SF/TF pair. Preferred orientation was defined as the phase of H2 [31]. By contrast, direction preference requires more careful analysis, as the phase of H1 alone is insufficient and known to yield biased estimates [30,38]. To overcome this, we reconstructed the direction tuning curve at each pixel by summing the tuning curves derived from both H1 and H2 components. Preferred direction at each pixel was then defined as the stimulus direction that maximized the resulting combined response. This approach yielded direction maps that closely matched those obtained using the standard technique based on episodic stimulation followed by Von Mises fitting [30].

The similarity index between two direction maps used in Fig 2D was estimated as follows. Consider two direction maps $\theta_{1}(x,y)$ and $\theta_{2}(x,y)$, where (*x*,*y*) are the coordinates on the cortex and that are associated to the same orientation map (that is, $\theta_{1}(x,y)-\theta_{2}(x,y)=0\text{mod}\pi$, is defined by the average number of pixels whose preferred direction differ by more than π/2:


$$\begin{array}{c} 1-\langle 1_{|\theta_{1}(x,y)-\theta_{2}(x,y)|\geq \pi /2}\rangle_{(x,y)\;\in \;\text{cortex}} \end{array}$$

**Fig 2:**
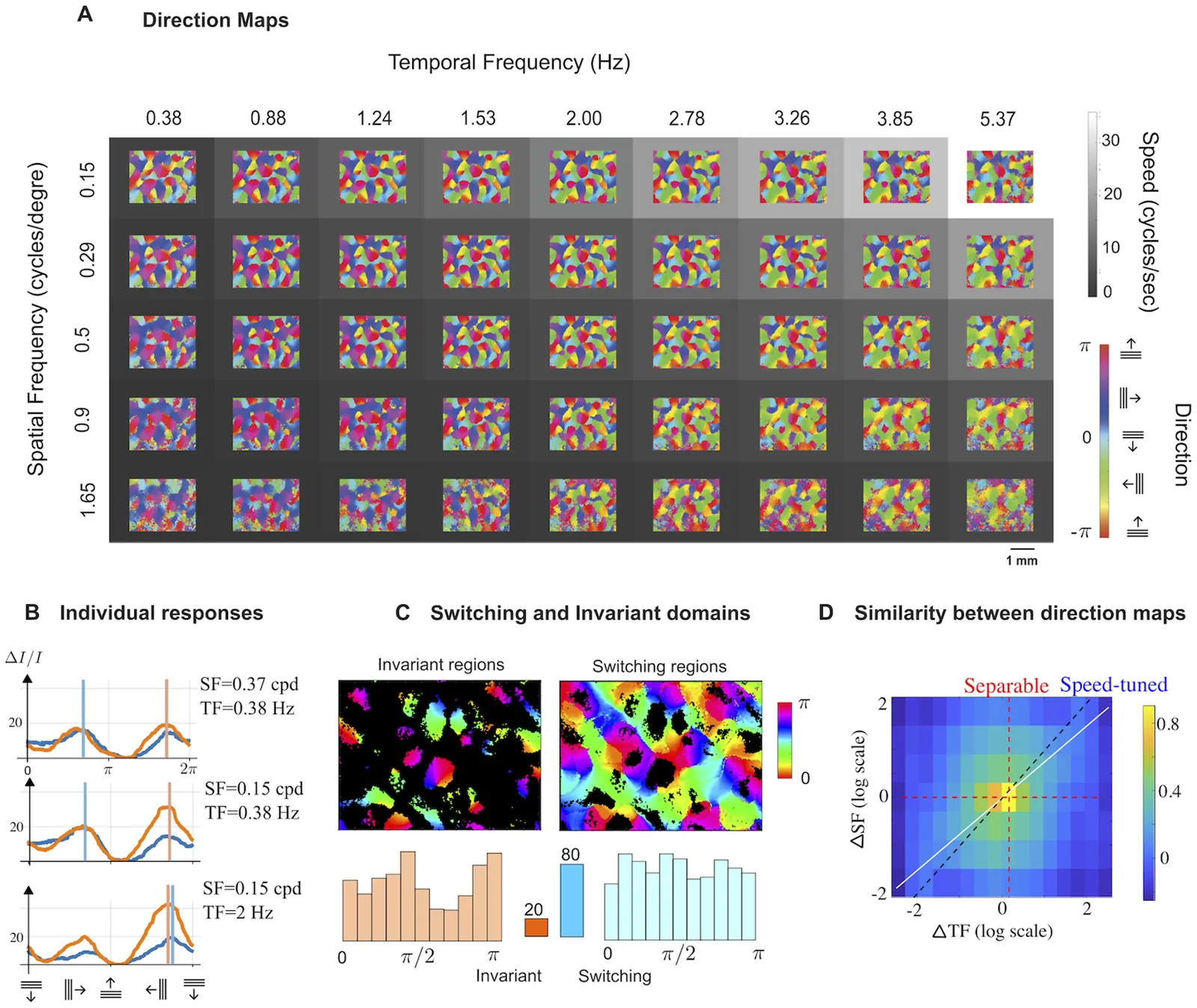
Variations of direction preference representation with stimulus temporal frequency and speed. **A.** Direction preference maps for all combinations of stimulus spatial frequency and TF,overlaid on a grey scale grid indicating stimulus speed. B Illustration of the direction tuning curves for two different cortical locations (blue and orange curves) and three different conditions (from top to bottom, (SF,TF)=(0.29,0.38), (0.15, 1.24), (0.15,2)). Vertical bars indicate the preferred direction of each tuning curve. **C.** Representation of cortical locations that do not switch direction preference (left, 19.97% of the cortical area) and those that do switch direction preference (right, 80.03% of the cortical area) overlaid on the invariant preferred orientation map. Histograms of preferred orientation are shown below, and these distributions exhibit no differences when corrected for continuity (*p* > 0.1, see test in Methods). **D.** Similarity index between pairs of direction maps, plotted according to the logarithmic distance between stimulus TF and spatial frequency. The axis of anisotropy is estimated using the model proposed in [4], with main axis shown as a white solid line, and, for comparison, the speed-tuned and separable axes indicated as dashed black and red lines.

where $\langle \cdot \rangle_{(x,yin\text{cortex}}$ denotes the average on all points of the cortical map, and $1_{condition}$ denotes the indicator function, equal to 1 when condition is satisfied and 0 otherwise. The choices of an indicator function and of a threshold π/2 are used to emphasize changes in the map that do not depend sensitively on the threshold. A test using the natural distance, $\langle \delta_{circ}(\theta_{1}(x)-\theta_{2}(x)rangle_{x\in \text{cortex}}$, where $\delta_{circ}$ is the circular distance $\delta_{circ}(\psi )=\text{atan2}(\sin(\psi ),\cos(\psi ))=\min(\psi \;\mathrm{mod}\;2\pi ,-\psi \;\mathrm{mod}\;2\pi )$, provides similar qualitative results.

### Fracture detection

To identify these fractures, we used an automatic extraction, identifying the maximal gradient in direction within a single map across x and y coordinates. In detail, we computed a discrete circular gradient of the direction map, for each value of SF and TF. To avoid creating artifacts due to the periodicity of the direction, we used a discrete gradient defined, for the partial derivative in the *x*-direction and at an interior point of the image, $\delta_{x}\theta (x,y)=\delta_{circ}(\theta (x+1,y)-\theta (x,y))$ where the circular distance is defined above. Because fractures are jumps of a fixed amplitude π, they largely dominate the gradient magnitude maps and stand out above noise and smooth regions. We thresholded these maps (at 3π/4), smoothed the fractures using mathematical morphology operations (a morphological closing followed by an opening, with a size of 1 pixel, using the imclose and imopen routines in Matlab, with a base element strel(’disk’,1)), and then skeletonized the elongated regions obtained into lines (bwskel method on Matlab). In parallel, pinwheels were detected using the π-circular gradient of the orientation map thresholded (with an adjustable threshold, by default chosen to be π/2) and then segmented using the Matlab routine bwconncomp, and pinwheels selected as the local maximum of the gradient in each region. A semi-automatic algorithm then displayed the pinwheels and the fractures together, and parsed the fractures for manual validation and refinement. Topologically, because pinwheels are periodic according to orientation (that is, π-periodic), each pinwheel is associated with a linear discontinuity in direction, called fracture. Mathematically, given the continuity of the direction map outside of fractures and pinwheels, fractures of direction cannot end at an arbitrary interior point of the map: they either reach a pinwheel or form a closed loop with no endpoints. While automatic classification of fractures is possible, we instead classified and refined the fractures manually. This step also served as a manual validation of each fracture derived and their refinements. We parsed each fracture identified, and had the ability to refine the shape of fractures. Each fracture was then manually classified it as a pinwheel fracture or a closed fracture; when identified as a pinwheel-connected fracture, the pinwheels closest to the ends of the fractures were added to the fracture. When identified as a loop, the two endpoints of the fracture were joined. Given the changes observed in the direction maps in Fig 2, we quantified the difference in fractures as a function of changes in TF and SF to compare the axis of change. Here again, measuring the distance between sets of fractures is a non-trivial task. To quantify the distance between the sets of fracture points associated with two conditions (hereafter, conditions 1 and 2), we proceeded as follows. For each fracture *F*_1_ in condition 1, we identified the fracture *F*_2_ which is closest to *F*_1_ (that is, the fracture for which the minimal distance between any two points of *F*_1_ and *F*_2_ achieves the minimal distance compared to other fractures $F_{2}^{\prime }$ in condition 2 - we used the Matlab routine pdist2 for this estimate). For fractures that connect two pinwheels, this typically selects the fractures that share the same pinwheel ends, except in rare situations of crossings where the two pinwheels of *F*_1_ are associated with two different fractures in condition 2, in which case one arbitrary minimal fracture was selected. Once the correspondence between *F*_1_ and *F*_2_ has been made, we estimated the distance between each point *x*_1_ of *F*_1_ and the fracture *F*_2_ (that is, the minimal distance between *x*_1_ and any point *x*_2_ on *F*_2_), and defined the *distance* between *F*_1_ and *F*_2_, $d_{frac}(F_{1},F_{2})$ as the mean of these distances over all $x_{1}\in F_{1}$ (note that taking the maximum would yield the Fréchet distance, which provided similar but noisier results). The distance between the sets of fractures in condition 1 and 2 was then estimated as the average distance computed over all fractures *F*_1_ in condition 1.

### Convolutional neural network

All Convolutional Neural Networks were implemented in Python 3 using PyTorch 1.12.1. Unless specified otherwise, they were trained on cropped images of size 256x256 pixels (i.e., $3.23\times 3.23\mu m^{2}$, for computational efficiency. All neural networks used shared the same architecture, composed of the following operations:

- Conv2d(1, 6, 5) + RELU: Convolutional step with 6 filters, that takes as input the reflectance, rescaled or difference N×N pixels data (these are single-channel images, thus the input size is 1) with *N* = 256 unless specified otherwise. The size of the convolutional kernels is 5 pixels (63μm). 6 filters are computed, resulting in 6 (N−4times(N−4) images. The results were rectified using RELU functions.
- MaxPool2d(2,2) reduces the dimension by taking, for each pixel, the maximum in a window of size 2×2 (yielding 6 output images of size (N/2−3)×(N/2−3))
- Conv2d(6, 16, 3) + RELU: the resulting 6 images are then subject to another 2D convolution with kernel size 3×3, and 16 filters, rectified with a RELU function. This results in 16 (N/2−5)×(N/2−5) images.
- AdaptiveAvgPool2d(1,1) takes each of the 16 outcomes and returns their average. This yields a 16-dimensional vector.
- Linear(16,120) + RELU + Linear(120,84) + RELU + Linear (84,1) the 16-dimensional vector is then processed through a series of 3 linear layers serving as a feedforward network to yield the regression.

Prediction error (or loss) was considered to be mean squared error, made periodic when predicting direction. When discussing and interpreting the results, shall refer to *decoding accuracy*, or simply *accuracy*, when comparing different prediction errors, which higher accuracy meaning lower prediction error. The optimizer was Adam (routine torch.optim.Adam), learning rate 0.001 by default (0.0005 for windows smaller than 20×20 pixels, or $255.2\times 255.2\mu m^{2}$). All results reported here are associated with such a regression algorithm. Classification for TF (where the output is a tensor of size 9 instead of 1 and loss is a traditional categorical classification accuracy associated with the percentage of accurate classification) yielded similar results. For smaller windows of size less than 20×20 pixels, we replaced the size of convolutional kernels by smaller kernels of size 3×3 pixels. Indeed, while kernels of size 5×5 pixels are useful to rapidly downsample larger images for easier processing, it is not necessary for smaller images where we can use more standard kernel sizes and maintain a sufficient number of input for our architectures to remain unchanged. Batch sizes were set to 32, and the number of epochs was set to 200, although convergence happened after around 100 epoch or less depending on the window size.

We used 10-fold cross-validation to generate our results. Controls were generated by using identical processes but shuffling the labels to be learned. Comparisons between the dataset and the controls were done using the corrected two-sample paired t-test, as proposed by Bengio and Nadeau [39], to handle the possible correlations between samples in a cross-validation experiment. Significance levels used were: ***: *p* < 0.001, **: *p* < 0.01, *: *p* < 0.05, and ns otherwise.

S4B Fig reports accuracies for simpler CNN architectures, described below:

**Model 0** Conv2d(1, 20, 5) + RELU + MaxPool(2,2)
+ AdaptiveAvgPool2d(1,1) + Linear(20,1)
  Model 0 is an elementary network based upon the same operations as the reference model above but with a single convolutional layer and a single linear layer. For more power, it also increased the number of convolutional layers to 20 instead of 6.
**Model 1** Conv2d(1, 20, 5) + RELU + MaxPool(2,2)
+ AdaptiveAvgPool2d(1,1) + Linear(20,30)+ RELU+ Linear(30,1)
  Model 1 is similar to Model 0, except that it has 2 linear layers for the regression, with dimension of the first layer’s input (and second layer’s output) 30.
**Model 2** Conv2d(1, 10, 5) + RELU + MaxPool(2,2)
+ AdaptiveAvgPool2d(1,1) + Linear(10,30)+RELU+
  Linear(30,50)+RELU+ Linear(50,1): This model now builds upon Model 2 and add a third linear layer, with output dimension of the second layer increased to 50.
**Model 3** Conv2d(1, 6, 5) + RELU + MaxPool(2,2)
  + Conv2d(6, 10, 5) + RELU + AdaptiveAvgPool2d(1,1)
  + Linear(10,1) Model 3 returns to two convolutional layers as the reference model (but with the second convolution having 10 filters) and reduces the number of linear layers to 1.)
**Model 4** Conv2d(1, 6, 5) + RELU + MaxPool(2,2)
Conv2d(6,16,3)+RELU + AdaptiveAvgPool2d(1,1) +
  Linear(16, 120)+RELU+ Linear(120,1) This model includes the same two convolutional layers as the reference model, but reduces the number of linear layers to 2, with the output dimension of the first layer equal to 120.

## Results

### Uniform TF tuning and invariant orientation preference

We began our investigation by examining how changes in stimulus motion properties (direction and TF) affected the reflectance activity in V1. For this purpose, we used a rotating and drifting sinusoidal grating [30,31] to probe responses in cat V1 across all orientations and directions, 9 temporal frequencies (ranging from 0.38 to 5.37 cycles per second) and 5 spatial frequencies (from 0.15 to 1.65 cycles per degree). Data were collected from three animals. Unless otherwise stated, all maps and examples shown in the main text correspond to animal 1, while corresponding analyses from the other animals are provided in the Supplementary Material.. We computed, for each condition, activity maps reflecting the level of activity of neurons at each given location and for each condition through the recorded hemodynamic signals, averaged over 10 repetitions of the stimulation.

We observed that patterns of activity displayed different responses upon changes in stimulus properties (Fig 1). Because we are interested in stimuli eliciting maximal responses as well as in the spatial reorganization of activity upon changes in stimulus properties, we decomposed the signals activity into two quantities: (i) the reflectance signal amplitude for a given condition (see Methods), and (ii) rescaled signals, that are linear transformations of the reflectance signal mapping their amplitude to [0,1]. Although in nature firing rate amplitudes are a function of a variety of properties of the stimulus (e.g., contrast and intensity) and attention mechanisms, it is classically considered that, within the controlled conditions of the experiment, changes in reflectance signal amplitude inform us of the features that elicit maximal firing rates overall. Rescaled signals instead provide information about the spatial reorganization of activity upon changes in stimulus properties. The data shows that varying the stimulus direction (Fig 1A, top row) caused major modifications in the spatial organization of activity, as visible in the rescaled maps but the signal intensity remained constant (right). In contrast, changes in TF did not result in major changes in the spatial organization of activity, but the intensity of the signal showed a clear dependence in TF, with a unimodal profile peaked at 1.53 Hz (Fig 1D).

The resulting preferred TF map depicting, for each pixel, the value of TF associated with a maximal response, did not exhibit spatial organization. This map indeed showed a globally spatially uniform dependence in TF and a majority of cortical locations (78–92%) exhibiting maximal responses for TF in [1.24,2.00] Hz (Figs 1C, and also S1A and S1B for two other animals). This uniform preference is of the one found in Bush Baby [27], and stands in sharp contrast with the spatial organization of other visual attributes, such as orientation or SF, where each cortical location marks a clear preference that spans a wide range of stimulus conditions [2,29,40]. Interestingly, variations in TF had no discernible effect on the representation of orientation (Figs 1D and S1C for two other animals). This map remained strikingly invariant with stimulus TF as quantified by the low median absolute deviation in preferred orientation upon TF variation (averaged across all cortical locations, mad = 0.04±0.02, 0.05±0.03, 0.03±0.02 rad for the three animals (in degrees, 2.29±1.15, 2.86±1.72 and 1.72±1.15), see S1D Fig). Altogether, this data argues for a uniform encoding of TF in V1 and no effect on preferred orientation.

### Smooth differential responses encode TF in V1

Despite this apparent uniformity in TF representation, we explored whether changes in activity could convey information about stimulus TF. To infer the presence of information on TF that could possibly be accessible to areas downstream of V1, we trained a convolutional neural network (CNN) to evaluate stimulus direction and TF from reflectance and rescaled responses. Similar approaches were previously applied not only to decode stimuli orientation [41,42] and direction [43] in humans, but also to investigate their representation with working memory [44–46] and mental imagery [47–50] and even to classify functional efficiency of aging cortices [51]. Moreover, these methods allow for a more detailed assessment of brain-activity patterns, revealing how even non-maximally responding regions contribute to decoding stimuli, as seen in previous studies [32].

Here, we found that the CNN was able to accurately decode both direction and TF from V1 signals with high levels of accuracy (low prediction error, Fig 1E), both from the reflectance signals and from the rescaled activity. The ability to decode the direction of motion from V1 activity is consistent with previous findings [43,52,53], and is expected, as strong activity shifts occur in response to changes in stimulus direction. However, given the low level of dependence of V1 response amplitudes on TF, particularly when rescaling maps of activity, it was unexpected to observe that TF could be accurately decoded from brain signals. This suggests that while the TF map is not organized into large-scale spatial patterns preferring various TFs, subtle yet spatially structured variations in the response maps still emerge and may encode TF information in a latent form accessible through advanced decoding methods.

To reveal these possible fluctuations, we computed the difference between rescaled activity maps at a given TF *t*, SF *s* and direction θ, noted A(t,s,θ), and the rescaled activity corresponding to the same SF and direction but a fixed *TF*, here chosen to be *TF*_1_ = 0.38*Hz*. The *difference maps*, defined as $A(t,s,\theta )-A(TF_{1},s,\theta )$, lose all the information about the rescaled responses, but revealed a strikingly organized structure that depends on TF, space, SF and direction (see Fig 1F, as well as S2 Fig for a more exhaustive representation of a set of differential responses as a function of TF and direction or SF). This rich and smooth structure uncovered through this operation, with amplitude and spatial organization showing progressive variation upon changes in TF, reveals a latent encoding of motion in V1. This spatial organization shows that, while the primary structure reflects a strong preference for direction, TF induces second-order fluctuations in the responses that could potentially carry information about the stimulus TF. To test this hypothesis, we trained a CNN to predict TF from the difference maps, and observed that TF was not only decodable from the difference map, but that accuracies was consistent with the reflectance images predictions (Fig 4E, squares, corrected paired t-test *p* > 0.05), indicating indeed enough information is contained in the differences to decode TF; similarly, we found that we could decode direction from response differences, with a small but significant (2-sided corrected paired t-test *p* < 0.001) decrease in accuracy (higher prediction error) compared to when it was decoded from the reflectance data. In S4A Fig, we further compared the learning speed of the CNN when trained on response differences or reflectance images. We observed that learning of TF was significantly faster when CNNs were trained on response differences, with fewer fluctuations and noise in the learning process, suggesting that response differences provided TF information that was more readily accessible compared to the reflectance images. These difference maps should not be interpreted as biologically accessible to downstream neurons, but helped identify novel structures in response maps that contains speed information. Moreover, these results did not depend on the choice of the reference TF used to compute the difference maps, as is expected based on the fact that changing the reference corresponds to a linear transformation of the data.

Going deeper into the structure of the signals, we considered the response amplitudes at four individual cortical locations for multiple directions and temporal frequencies (Fig 1G, smoothed to reveal their structure, see methods). Remarkably, we found that while most directions considered were associated with unimodal responses peaked around the common preferred TF, the dependence in TF of response amplitudes showed distinct profiles between different directions at the same location. In Fig 1G, we depicted responses at the preferred and opposite direction, as well as responses associated with a direction perpendicular to the preferred direction. All three curves showed vastly distinct profiles, which allows the emergence of complex structures in the response difference while maintaining globally uniform preferences in TF and unchanged first-order responses. Furthermore, Fig 1G also confirmed, consistently with previous work [7,28], that specific locations may change preferred direction according to TF (black triangles in Fig 1G), an effect we explored in more detail in subsequent sections.

Altogether, these findings show that while we found that the preferred TF map is globally uniform, the minute changes in amplitude with stimulus TF differ from location to location, suggesting that V1 encodes more nuanced information about TF than previously recognized.

### Continuous changes in direction maps fractures

We further explored the structure of changes in direction preference by reconstructing and analyzing preferred direction maps for each combination of temporal and spatial frequency tested. We observed substantial variations in direction maps upon variation of spatial and temporal frequency (Figs 2A and S3 for the other two animals), with a mean absolute deviation tripled compared to that associated with orientation maps (0.12 + 0.11 rad, 0.17 + 0.13 rad, and 0.09 + 0.09 rad for the three animals). These variations in direction maps closely align with changes in direction tuning curves upon TF variation, as suggested by single-cell studies in ferret [7,28] and exemplified for two cortical locations for different values of stimulus SF and TF (Red and blue curves, Fig 2B). Moreover, the spatial location of these shifts in preferred direction were not confined to the vicinity of pinwheel centers (Figs 2C and S3C for the other two animals). Instead, large regions of the cortical surface switched their preferred direction (Cat 1: 80.23%, cat 2: 59.20%, cat 3: 77.00%), with no significant bias in the preferred orientation of the regions that switch direction preference (Rao test for uniformity, corrected for continuity of the maps; see Methods).

The matrix layout of the different maps in Fig 2A additionally suggests that changes in direction maps are not separable when stimulus SF or TF are varied independently. Instead, the changes seem dependent on a linear combination of the two stimulus attribute. This apparent anti-diagonal invariance was evocative of the linear relationship that was fitted to describe the joint TF and SF tuning of single cells in [4]. In that paper, the authors investigated whether single-cells tuning for TF and SF was separable or if it depended on a power-law relationship between preferred TF at each SF, with in particular the aim of testing whether cells could be tuned for speed, TF/SF. The stimulus speed is depicted as the greyscale background in Fig 2A. To estimate the axes of maximal sensitivity in direction maps upon changes in SF and TF, we quantified the similarity of direction maps across all conditions. We found that maximal similarity between maps was achieved along a linear axis in the logarithmic scale of TF and SF differences. By fitting the Gaussian function proposed in [4] over a range up to 4.6 octaves of SF and TF differences (i.e., $\frac{TF_{1}}{TF_{2}}$ and $\frac{SF_{1}}{SF_{2}}<25$), we identified that the main axis of the fit followed the line log(TF)≈0.76log(SF) (or ξ=0.76), see Fig 2D. This relationship reflects an intermediate scaling between pure speed and pure TF (cat 2, ξ=0.62; cat 3, ξ=0.72; see S3D Fig) which was confirmed computing the bootstrap confidence intervals (S5 Fig) evocative of the tuning properties of single cells [3,4,21]. While the value obtained for the stability of the map was typically less variable (S5 Fig) and suggesting that a similar relationships holds, at the level of neural populations and in terms of functional representations in the direction map. Overall, our findings indicate that there are co-organized changes across the majority of the direction maps as TF and SF vary, following an axis between pure-TF and pure-speed tuning. This suggests that V1 direction maps integrate information from both TF and SF in a coordinated manner, tuned to a combination of speed and TF.

### Fractures remain stable for constant stimulus speed

Modulo π, direction preference maps share the typical organization of orientation preference maps, which are continuous except at orientation pinwheels - punctual singularities around which all orientations are represented. Due to this constraint, direction maps are bound to display linear singularities, called fractures, where direction preference displays a sharp discontinuity, shifting to the opposite preferred direction (Fig 3A). Topologically, each pinwheel is associated with at least one fracture. Typically, direction fractures consist of linear singularities connecting two pinwheels or forming closed loops.

**Fig 3:**
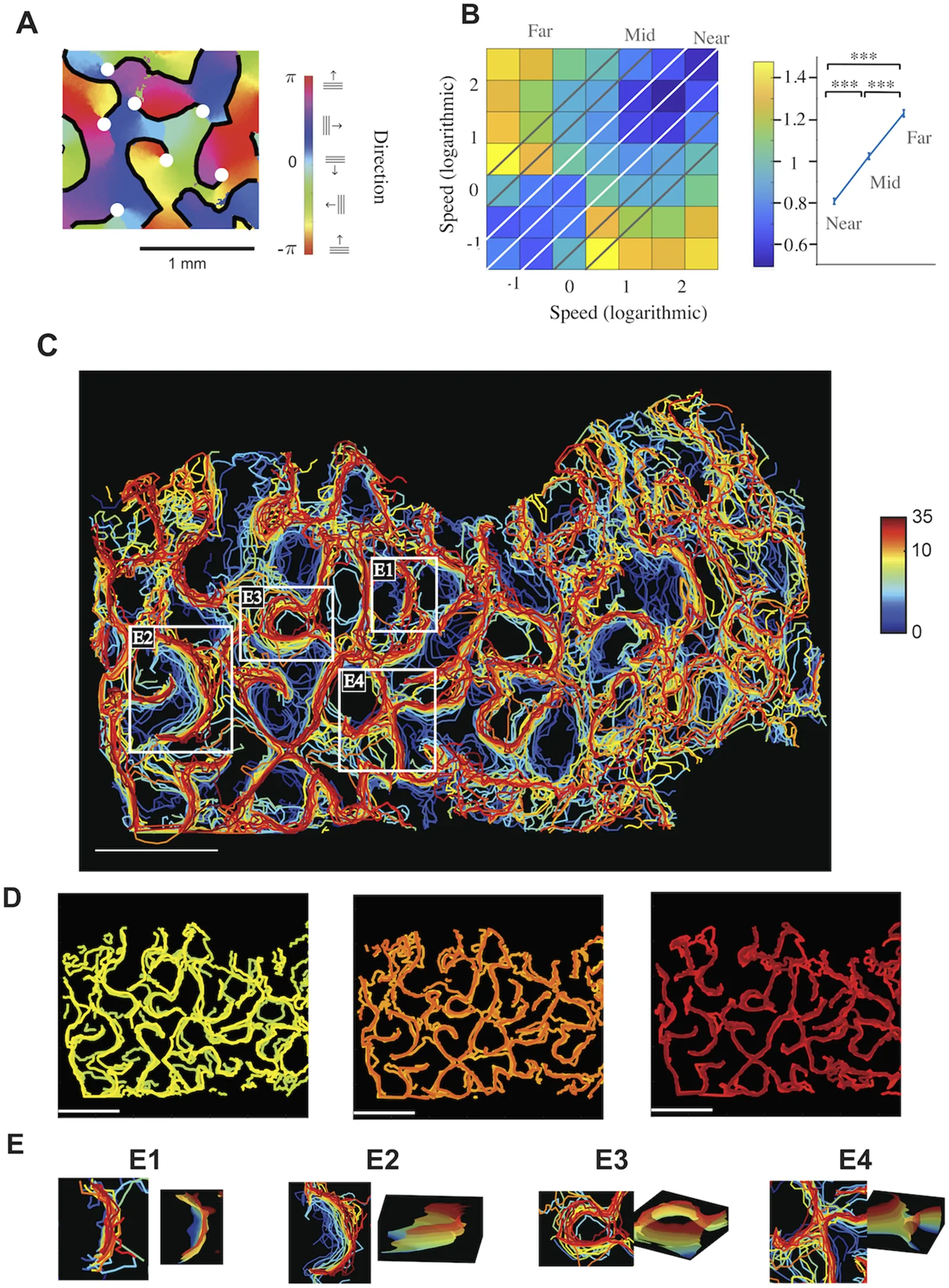
Shifts in direction maps fractures with speed reveal stereotyped motifs. **A.** Illustration of preferred direction map fractures (black contours) overlaid with orientation pinwheel centers (white dots) for *TF* = 1.53 Hz and SF = 0.29 cpd. **B.** Average distance between fractures in pairs of direction maps, plotted according to the speed (in logarithmic axes), suggesting that the distances increase with speed difference, which is confirmed in the graph on the right showing the mean and sem of the distances for similar speeds (’Near’, which include the three diagonal bins,’Mid’ that include the next two bins, and the rest is pooled in the’Far’ statistics, 2-sample t-test, *p* < 0.001). **C.** Direction fractures computed for each combination of stimulus TF and spatial frequency, colored-coded according to speed (in logarithmic scale). **D.** Representation of direction fractures across different speed intervals, where minimal changes occur as shown in B: left panel shows speeds in [2.45,5.5] degree/second (7 conditions), middle panels in v∈[6,14] degree/second (7 conditions), and right panel in v∈[14,33] degree/second (5 conditions). **E.** Illustration of typical fracture movements with speed, shown as a zoomed-in view from C with a 3D surface representation where the **(*x*,*y*)**-axes represent the cortical surface and (*z*-axis) represents speed. E1: Invariant direction fractures. E2: Traveling fractures, where fractures shift in one direction with speed. E3: Bubbling fractures, where one fracture splits into two, with one forming a closed loop. E4: Crossings fractures, where two fractures exchange their connections.

To investigate changes in direction preference and potential topological changes in direction maps, we meticulously tracked direction fractures in each of the 45 direction maps (9 TFs × 5 SFs, Figs 3C and S3A for the other two animals). These fractures exhibited continuous variations in response to changes in speed, covering large regions of the cortical map. To quantify changes in fracture positions, we computed an estimate of the distance between sets of fractures (see Methods) as a function of stimulus speed. We observed that the greatest invariance occurred along the identity axis, suggesting indeed that minimal changes in the position of the fractures arise between maps associated to similar speeds, and when speeds differed, the fractures moved away from each other (Fig 3B). This invariance became even more apparent when fractures were grouped according to speed: positions of fractures showed only minor variations for stimuli associated with similar speeds, even when SF and TF differed significantly (Fig 3D).

As the stimulus speed changed, some fractures remained spatially stable (Fig 3E1), while some others were gradually shifting, in a majority of cases moving along a given axis maintaining the identity of the pinwheels they are connected to (Fig 3E2). Occasionally, fractures formed detached closed loops (bubbling, Fig 3E3), or exchanged which pinwheel they were connected to (saddles, Fig 3E4). Of note, similar instances were found in all animals considered (see S3B Fig, right). These topological changes actually arise from the continuous morphing of the fracture shapes, as illustrated in the three dimensional reconstructions of the fracture surfaces, where speed is represented as altitude. Altogether, these findings reveal that direction fractures in V1 display major topological modifications upon variation of stimulus speed, resulting in reversals of preferred direction across large portions of the cortex, but remain largely stable for stimuli with similar speeds.

### Local decoding of speed from V1 activity

The observation that direction maps are not unique, but vary with speed, raises questions about the possibility of decoding the direction of stimuli with distinct speeds, as well as the independent decoding of speed and direction. To explore this, we used a similar convolutional neural network as in Fig 1E) and trained a regression algorithm to predict speed from reflectance or rescaled data (Fig 4A). Remarkably, we found that reflectance (Fig 4B) or rescaled (Fig 4C) brain activity images produced highly accurate speed and direction predictions, with performance significantly better than control datasets where the association between brain activity images and speed was randomized. To assess whether this decoding performance required a deep or complex network, we tested multiple CNN architectures with varying levels of complexity (S4B Fig). All tested architectures, including those with minimal convolutional and linear layers, were able to extract stimulus information significantly above chance, confirming the robustness of the observation of decodability of speed from the activity maps, irrespective of the specific parameters of the algorithm used. Furthermore, we found that speed and direction information can be decoded locally, in cortical regions as small as $123\times 123\mu m^{2}$ (10-by-10 pixels with our resolution) (Fig 4C). The algorithms used to decode local activity had the same architecture, with the only difference being the size of the convolution kernels, which were reduced from being 5×5 pixels to 3×3 for regions smaller than 20× 20 pixels, see Methods. Even in these small regions, the decoding accuracies were comparable to that of larger areas, suggesting that speed encoding is a local property of V1 responses. This may imply the animal’s capacity to detect complex movements associated with various speeds and directions at different locations in the visual scene.

**Fig 4:**
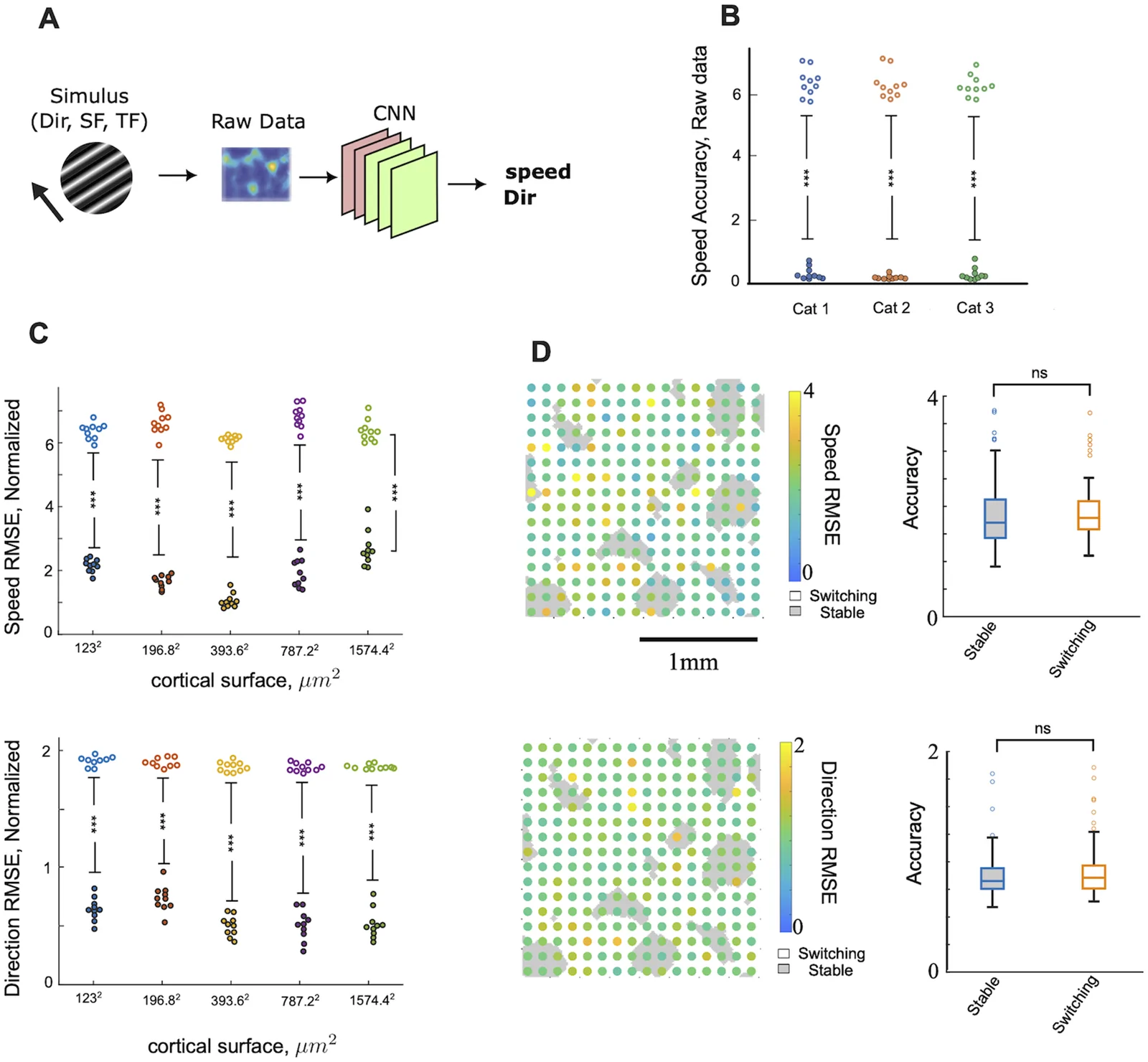
Preferred direction and preferred speed are accurately decoded from local V1 responses despite uniform or variable representation. **A.** Illustration of the convolutional neural network architecture. It is composed of five layers: two convolutional layers (pink) and three linear layers (green), with rectified linear activation functions. **B.** Prediction error for speed accuracy across three animals shows high predictability compared to controls (same dataset but where TF and Direction were randomly shuffled) (10-fold cross-validation, two-sample t-test *p* < 0.001). **C.** Prediction error for speed (top) and direction (bottom) across cortical regions with different size (from $123\times 123\mu m^{2}$ to $1574.4\times 1574.4\mu m^{2}$), shows high predicability compared to controls (same dataset but where TF and Direction were randomly shuffled) (10-fold cross-validation, two-sample t-test *p* < 0.001). **D.** Spatial organization of coding prediction error for preferred speed (top) and preferred direction (bottom) of non-overlapping cortical windows with 10×10 pixels ($123\times 123\mu m^{2}$, colored circles) overlaid with invariant (grey) and switching (white) direction domains. Right panels show no significant difference in prediction error between invariant and switching direction domains (2-sample t-test *p* > 0.1).

Finding that speed was decodable at local scales thus allowed the investigation of potential correlations between cortical position in V1 and speed decoding accuracy. To do that, we computed on a fine grid of non-overlapping regions of $123x123\mu m^{2}$, speed and direction decoding accuracy over a large area of V1 (Fig 4D, left panels, top for speed and bottom for direction). While there were some spatial fluctuations in accuracy, no clear correlation emerged between these spatial fluctuations and changes in preferred direction with stimulus speed. To quantify this observation, we labeled each window as either “switching” or “stable” depending on whether the majority of pixels contained in that area changed direction preference with speed. Notably, we found no significant difference in decoding accuracy between these two regions (Fig 4D, right panels). This suggests that the spatial variations in direction preference and speed decoding accuracy are two sides of the same coin, the differential changes in response amplitudes rather than consequences of one another. Also, no correlations were found between the coding accuracies in speed and direction.

## Discussion

Our findings provide new insights into how motion attributes are represented in V1 and their potential implications for visual perception. We demonstrated that TF preference is uniformly distributed across V1, lacking spatial modulation typical of other visual attributes. Despite this apparent invariance, we found that TF information could be decoded from raw signals, suggesting a *latent encoding* of temporal frequency in the sense of information, meaning that TF information is not manifest in the patterns of activity but is present in the form of subtle but organized changes in the responses that we revealed through the calculation of difference maps. These subtle differences also significantly affected the organization of direction preference exhibits significant remapping in response to changes in TF and speed, with about 60–80% of the cortical area switching preferred direction under varying conditions, putting in perspective the notion of a well-defined direction map in V1, but revealing a collection of maps smoothly varying upon changes in TF. In sharp contrast, the OR map was invariant to changes in TF, revealing that some maps are robust to changes in a given attribute while others are variable. Here again, despite this variation in direction maps, the changes in cortical activity associated can provide fine and local information about the direction, as we showed using convolutional neural networks (CNNs) to decode motion attributes from brain signals, including TF, speed, and direction. These results provide evidence consistent with the hypothesis that maps organized according to stimulus preferences covering the range of percepts, or even representations of visual attributes that are invariant upon changes in other stimulus properties, are not necessary for visual perception. This offers a novel perspective on how V1 may encode motion attributes. Despite the limited number of animals used in the present study (N = 3), these qualitative observations were consistently found across all animals. Nevertheless, this sample size limits our ability to assess the full extent of inter-animal variability and the generalizability of quantitative estimates.

Pioneering investigations of TF representation in cat V1 have reported the presence of binary domains with a co-representation of SF: regions responding preferentially to low SF and high TF or high SF and low TF [26]. While subsequent studies have revealed that SF representation in cat V1 spans a broad range of preferences and is predominantly continuous [29,40], the case of TF representation has not been reexamined. Here, we demonstrate that TF preference in V1 is mostly uniform across the cortical surface, with a 80–90% of cortical locations exhibiting maximal responses in the 1.24-2 Hz range, and lacking the spatial modulation suggested by earlier studies. These findings align closely with results obtained in promisian primates Bush Babies [27], where TF preference was similarly reported to be uniformly distributed, centered around 2 Hz. Additionally, TF had a significant impact on the representation of direction preference. An organization for direction preference in cat V1 has been reported [6,8,38], but these studies examined responses to a single TF of stimulation. Our data reveal that direction map is not invariant with respect to stimulus TF and that TF and direction should be studied together. Interestingly, these shifts in direction preference with stimulus TF align with specific map motifs, suggesting an underlying structure to this variability.

Our study relied on highly controlled drifting gratings that dissociate TF, SF, and direction. This approach had the advantage to allow shed new light on the uniform coverage hypothesis, a foundational principle of cortical organization [54] postulating that the different attributes are encoded into parallel maps that favor even representations of combinations of attributes [55–57]. Two of our findings challenge the uniform coverage hypothesis for motion-related features: (i) the predominantly uniform distribution of TF preference contradicts the expectation of even representation across the attribute’s full range, and (ii) the dynamic changes in direction preference with stimulus TF violate the principle of invariant representation. These observations suggest that uniform coverage may not extend to motion-related features and that alternative mechanisms might govern their cortical representation. Cortical responses to naturalistic stimuli are however known to differ substantially from those evoked by drifting gratings in various contexts. In particular, natural movies can induce distinct temporal response dynamics and higher temporal reliability in cat V1, sparser [58,59] and may reveal response properties that are less apparent under artificial stimulation [60,61] or code for second-order statistics while preserving robust tuning in subsets of neurons [62]. An important future direction will be to determine whether similar dynamic reorganizations of direction-related activity emerge during stimulation with natural movies or dynamic naturalistic textures.

Our CNN analysis provides compelling evidence that functional maps in cat V1 are not necessary for the perception of motion attributes. Indeed, despite the lack of a structured TF map and the variations of direction maps, we demonstrated that TF, speed, and direction information could be accurately decoded from local V1 responses at any cortical location. Whether this information is indeed decoded in areas downstream of V1, and the putative mechanisms by which this information would be extracted, are still unknown. But it remains that information is present in the activity patterns of V1, so, theoretically, accessible. Another important perspective of this study is to better understand how and where the information on speed is encoded in V1. In that view, we investigated various modifications of the dataset, including normalizing signals, computing the difference between normalized signals, localizing the stimulus, and did not find a specific pattern or motif governing the coding accuracy in the network. Similarly, we found no correlations between fracture movement and coding properties. Identifying how the information is coded therefore remains an open problem and further experimental or theoretical analyses on the CNN, such as fine investigations of the saliency maps, constitute exciting avenues for future research. Along the same lines, whether the motion-related information revealed here reflects computations performed locally within V1 or information inherited through recurrent interactions with higher visual areas remains an open question. Importantly, however, both possibilities are compatible with the presence of motion-related information in V1 population activity and with its decodability from cortical responses. The question of the functional significance of maps for visual perception echoes findings in squirrel monkeys, where, variability in the expression of ocular dominance columns -from well-developed to nearly absent- does not impair binocular vision [63,64], challenging the presumed necessity of these maps for stereopsis. While visual systems across animal species are quite different, this observation is consistent with the observation that rodents [6,65], lagomorphs [66] and grey squirrels [67] are able to maintain robust visual processing without the columnar organization of orientation or direction, already underscoring that maps may not be indispensable for perception. Instead, we suggest that distributed activity patterns in V1 may convey rich information about motion attributes.

How, developmentally, these co-representations arise remains a central question. Mathematical or computational models, capable of successfully reproducing different aspects of V1 response properties [68–75], may collectively suggest that plasticity and the nature of visual stimuli underlie map organizations. In particular, models based on the statistics of natural images and their processing [76–80] or neurogeometric models [81–83] tend to suggest that geometric structures and the nature of stimuli are the source of functional architectures. An intriguing avenue of research would be to explore these models for speed invariance, fractures. Some of our topological observations and rules of change arise naturally from biological constraints, such as the co-organization of orientation and direction tuning, and stability of orientation tuning across TF changes. Testing if models of V1 and visual perception generate similar responses to varying TF and speeds offers an interesting perspective of this work.

Consistent with physiological perception, the stimulation we used was binocular, which does not allow us to infer whether the structures observed emerge from binocular interactions or if they are the result of the superposition of the input received by both eyes. Further experiments will be necessary to test this by stimulating one eye at a time, which would require adapting the experimental protocol to accommodate possible longer experimental times.

Intrinsic optical imaging signals are indirect measures of neuronal activity, reflecting the impact of neural spiking on localized changes in blood oxygenation and volume. Because it provides access to mesoscopic signals over relatively large cortical areas for extended periods, it is well-suited for large-scale mapping of functional organization. Its spatial and temporal resolution is, however, limited, and typically prevents inferring precise patterns of single-cell activity. A significant advance was made in understanding the relationship between hemodynamic signals and neural activity, particularly in the physiology of neurovascular coupling that underlies intrinsic optical imaging and functional magnetic resonance imaging (fMRI). Fine experiments highlighted the correlation between spiking vascular activity [84] and even revealed the existence of orientation selectivity at the level of individual blood vessels in cat V1, but also that spiking and hemodynamic signals are partially decoupled. Moreover, the consistency between hemodynamic signals and spiking activity depends on the cortical layer and on independent anatomical constraints, such as vessel anatomical orientation through the cortex and capillary organization. Signals were also found to be transported through these vessels to distant regions of the cortex [85,86]. All of these observations are fundamental limits to the resolution at which hemodynamic signals can reflect local spiking activity. This motivates combining, in future studies, optical imaging techniques with single-cell resolution techniques to further understand how spiking activity supports the changes in responses we reported, and to assess consistency between neuronal responses and speed selectivity at the single-cell level.

The combination of beautiful and rule-bound topological changes with the co-organization of response properties to all four stimulus properties (OR, DIR, TF, SF), naturally leads to developmental and structural questions. Developmentally, we know that direction selectivity requires visual experience in carnivores [87,88] and primates [89], so it is of interest whether co-organization with speed tuning, in particular, follows a strict developmental ordering or is influenced by experience. Further, direction maps and orientation maps are co-organized spatially in columns about pinwheel centers, and given the finding that direction maps change with TF /speed and orientation maps remain constant a natural question arises of whether there is a hierarchy of priority and stability in V1 feature processing, with a strong robustness of orientation tuning invariance, and whether this stability coincides with bottom-up versus top-down activity modulation. The prior segregation of motion processing to higher-order regions, and stationary processing to V1 followed a bottom-up theory of building complexity in processing, but the recurrent connections from these “higher-order regions” in visual processing back to V1 calls into question the extent of the observed motion information arising as a consequence of “top-down” information transfer [10]. One approach to addressing this question is investigating direction map changes under conditions of targeted optogenetic/chemogenetic silencing of projections to V1 from regions downstream.

## Supporting information

S1 Fig Optical imaging responses in cats 2 and 3 (A) Rescaled signals as a function of TF and (B) histogram of preferred TF (analogous to Fig 1C), (C) Orientation maps as a function of TF and (D) their pixel-wise standard deviation (C-D compare to Fig 1D). This confirms the observations made in cat 1 of a uniform representation of TF in V1 with a peaked distribution and an overall invariance of orientation maps as a function of TF.
(TIFF)

S2 Fig(A-B) Difference activity maps for various directions and temporal frequencies (SF = 0.29 cycles/degree), with a reference map of TF = 0.38 (scalebar from dark blue to dark red with, for A: a fixed difference scale clipped at [-0.5, 0.5] and white for 0), and for B: same data, but each figure is individually scaled in [0,1].The colorbar grows from 0 to 1, with 0.5 corresponding to white. C. Difference maps as a function of SF and TF with fixed direction π/2 rad, represented with a common scalebar clipped at [-0.2,0.2]. Similar to Fig 1F, we observe that the difference maps are highly structured and reflect fine changes in activity upon variation of TF, SF and direction.(TIFF)

S3 FigChanges in direction preference, cats 2 and 3.(A) Changes in direction preference as a function of TF and SF for cats 2 and 1 (for cat 1, see Fig 2A) and (B) location of the fractures as a function of speed (color) as well as 4 examples of the types of fracture movement (for cat 1, see Fig 3C and 3E). (C) represents the preferred directions in the invariant or switching regions (Fig 2C for cat 1 analyses). (D) represents the similarity between direction maps as a function of the logarithmic difference in SF and TF, with a Gaussian fit (ellipses and vectors) in $\log\left(\frac{sf}{sf_{0}}\right)$ and $\log\left(\frac{tf}{tf_{0}}\right)$ within 2.5 octaves. Significant linear relationships with respective slopes 0.84, 0.78 and 0.94 were found, with a ratio of excentricities $\frac{\lambda 1}{\lambda 2}$ of, respectively, 1.63, 1.47 and 1.52.(TIFF)

S4 FigStimulus decoding.(A) Accuracy of speed decoding for a CNN trained with difference maps. (A1) Significant learning is achieved compared to control (10 fold cross validation, 2-sided t-test *p* < 0.001). (A2) Show validation accuracy as a function of learning epochs (32 learning steps) for the CNN trained with difference maps (blue) or with the reflectance maps (red) for each of the 10 folds of training. Learning from difference maps is faster and more consistent, while learning from the reflectance maps is slower, more variable, and subject to local minima. (A3) shows a statistical analysis of these curves (mean ± sem), with the p-value level associated to a 2-sample t-test for each epoch plotted in color in the background. We can see a significant difference at initial epochs, confirming the fact that learning is significantly faster when training is done for the difference maps, but final accuracies show less difference, and, at the final epoch, no significant difference is found. (B) and (C) are analogous to (A3), but correspond respectively to the learning of direction and TF. Similar, yet less pronounced, phenomena happen for learning of direction and TF, with however a significant degradation of learning direction when training with difference data. (D) Speed decoding accuracies on 5 models with increasing degrees of complexity (see Methods for description of the Models). Blue: decoding accuracy (MSE), compared to control (orange). All models show significant learning (*p* < 0.001). Data used: cat1.(TIFF)

S5 FigSimilarities between direction maps of cats 1, 2 and 3 according to the logarithmic differences $\Delta SF=\log\left(\frac{sf}{sf_{0}}\right)$ and $\Delta TF=\log\left(\frac{tf}{tf_{0}}\right)$, spaced into 7 and 17 values respectively.Top: average similarity map for each fixed logarithmic difference, together with a fit of the Gaussian model proposed in (Priebe et al, 2006) within 2.5 octaves. Significant linear relationships with respective slopes 0.76, 0.62 and 0.72 were found, represented as the white line in the graph, with, for comparison, axes for separable tuning (red dashed) and speed tuning (black dashed). (B) Bootstrap confidence intervals show that the estimate for ξ obtained are robust and vary very little – in particular, the distribution does not include separable or speed-tuning regimes. (C) coefficient of variation of the similarity index in each bin, showing a small level of variability.(PDF)
