# Supplementary material for: Latent encoding of movement in primary visual cortex
Source: PLoS Comput Biol. 2026 Oct 12;22(10):e1014826. doi: 10.1371/journal.pcbi.1014826 (PMC13712730; doi:10.1371/journal.pcbi.1014826)
Supplement: Supplementary file 1 [file pcbi.1014826.s005.pdf]

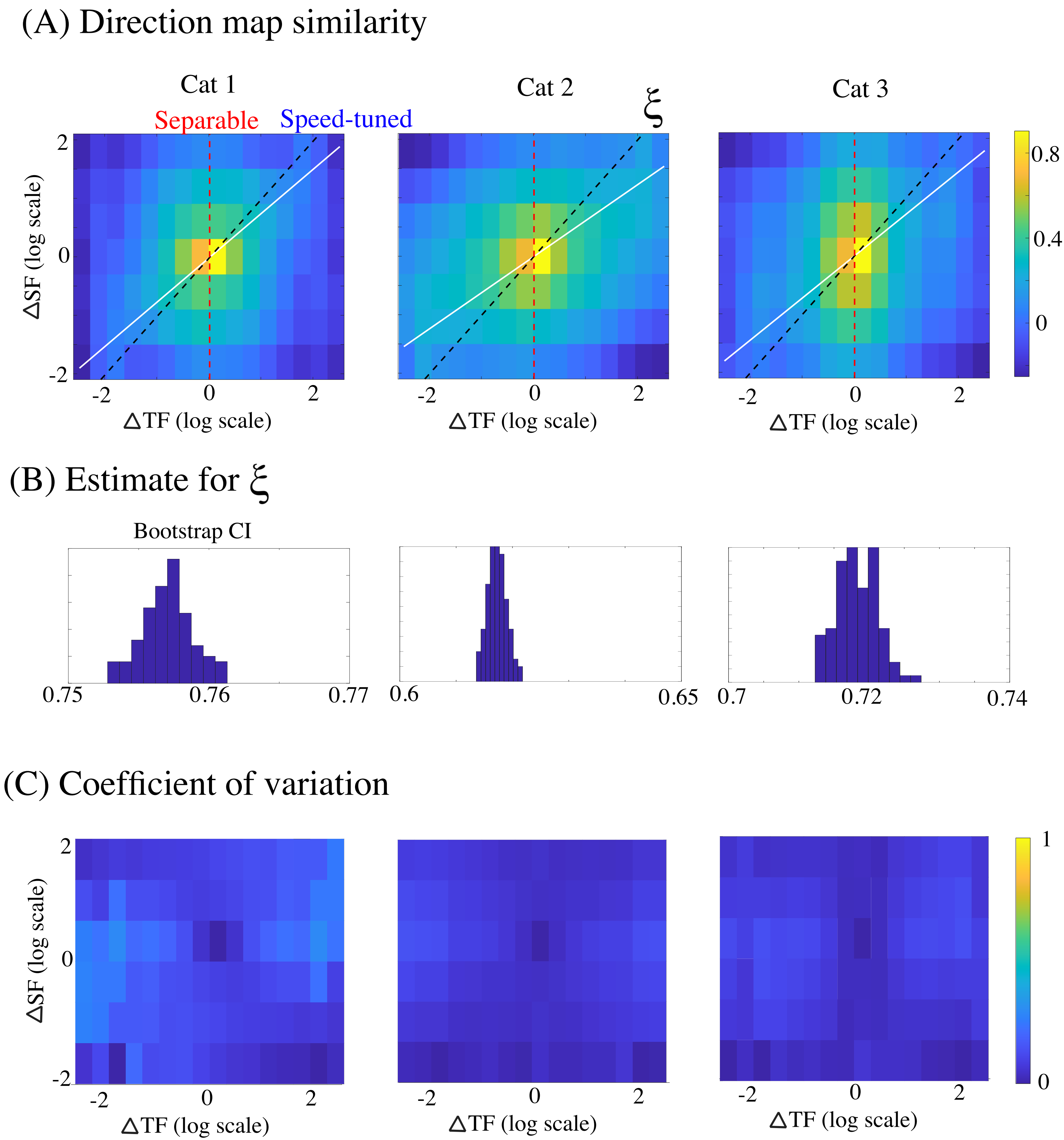

(A) Direction map similarity
Cat 1
Cat 2
Cat 3
Separable
Speed-tuned
ξ
ΔSF (log scale)
ΔTF (log scale)
0.8
0.4
0
(B) Estimate for ξ
Bootstrap CI
0.75
0.76
0.77
0.6
0.65
0.7
0.72
0.74
(C) Coefficient of variation
1
0
